# Increase in complicated upper respiratory tract infection in children during the 2022/2023 winter season—a post coronavirus disease 2019 effect?

**DOI:** 10.1007/s00247-023-05808-1

**Published:** 2023-11-24

**Authors:** Corona Metz, Andrea Schmid, Simon Veldhoen

**Affiliations:** Charité – Universitätsmedizin Berlin, corporate member of Freie Universität Berlin and Humboldt-Universität Zu Berlin, Pediatric Radiology, Augustenburger Platz 1, 13353 Berlin, Germany

**Keywords:** Computed tomography, Coronavirus disease 2019, Pandemic, Pediatrics, Upper respiratory tract infection

## Abstract

**Background:**

Upper respiratory tract infections usually peak during winter months.

**Objective:**

The purpose of this study was to evaluate whether imaging of complicated upper airway infection in children increased during the winter season of 2022/2023.

**Materials and methods:**

In a retrospective study setting, pediatric magnetic resonance imaging (MRI) and computed tomography (CT) scans for evaluation of upper respiratory tract infection performed between October 2022 and April 2023 were analyzed regarding presence of the following complications: mastoiditis, abscess, phlegmon, meningitis, reactive vasculitis, and sinus vein thrombosis. Pathogen detection, surgery, and infection parameters were obtained. Data were compared with MRI and CT scans performed in the same months of the preceding five years, distinguishing between pandemic and pre-pandemic years.

**Results:**

During the 2022/2023 winter season, the number of MRI and CT scans in children with upper airway infections, the complication rate, the rate of detected streptococcal infections, and the rate of surgery increased significantly compared to expectations based on the five prior winter seasons (all *P*<0.05). During the first complete pandemic winter season in Europe (2020/2021), the number of MRI and CT scans in children with upper airway infection, the complication rate, and the rates of streptococcal detection and surgery decreased significantly compared to expectations based on the pre-pandemic, the second pandemic, and the post-pandemic winter seasons (all *P*<0.05).

**Conclusion:**

After a decline during the first pandemic winter season, there was a marked rebound in complicated upper airway infection in children, with a significant increase in cases during the 2022/2023 winter season compared to the pre-pandemic and pandemic years.

**Graphical Abstract:**

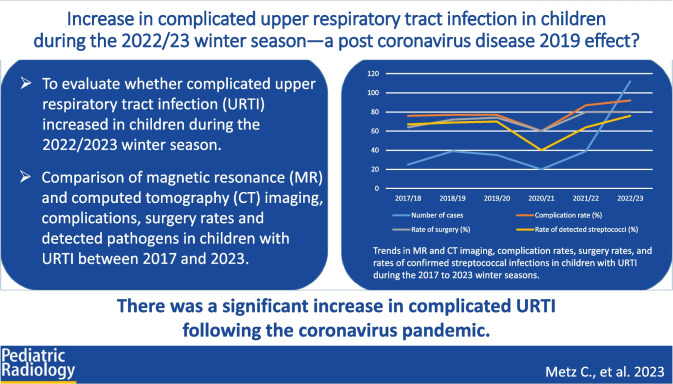

## Introduction

In January 2020, the first cases of severe acute respiratory syndrome coronavirus 2 (SARS-CoV-2) infections were reported in Europe and in March of 2020, the World Health Organization declared a global pandemic [1, 2]. Especially in the first year of the pandemic, there were strict measures such as quarantine, face masks, and distance regulations, to prevent respiratory tract infections and hospitalizations [3]. These actions did not only limit the transmission of SARS-CoV-2 virus, but simultaneously interrupted person-to-person transmission of many other pathogens typically causing pediatric respiratory tract infections, such as *Haemophilus influenza*, *respiratory syncytial virus*, and *Streptococcus pneumoniae* [4–7]. Subsequently, declining numbers of infectious disease diagnoses during the coronavirus disease 2019 (COVID-19) pandemic attributable to the above measures were noted in many countries [8–17].

Countries chose very different measures to cope with the pandemic. Some maintained strict isolation for very long periods of time once regional outbreaks occurred (“zero COVID” strategy, e.g., China) [18], others adapted their actions to regional incidences (e.g., Germany) [19], and some countries took minimal action (e.g., Sweden) [20]. Economic and social costs of the mentioned measures have been significant, leading many countries to loosen their restrictions over time. After three years of the COVID-19 pandemic, in April 2023, the pandemic was officially considered over in Europe and restrictions were withdrawn.

Upper respiratory tract infections show a strong seasonality and usually peak during the winter months [21]. In the first post-pandemic winter season 2022/2023, we observed a marked increase in the number of magnetic resonance imaging (MRI) and computed tomography (CT) examinations in children with upper respiratory tract infections and an increase in serious complications.

Therefore, the aim of the study was to analyze whether there was a statistically significant change in complicated upper respiratory tract infection before, during, and after the COVID-19 pandemic, based on imaging data from a large university pediatric radiology department in Germany.

## Material and methods

### Institutional review

The study was approved by the institutional review board.

### Study sample and design

Children, who had clinically indicated MRI or CT scans of the upper airways (head, face, and neck) for the evaluation of complicated upper respiratory tract infections between October 2022 and April 2023, were included in this exploratory study. Imaging protocol and MRI sequences were selected according to departmental standards and the respective clinical question. MRI examinations were acquired on a 1.5- or 3-tesla scanner (Siemens, Erlangen, Germany) and included T2-weighted and diffusion-weighted sequences as well as T1-weighted images before and after administration of contrast agent. CT scans were acquired on a 128-detector scanner (GE Healthcare, Chicago, IL) or a 320-detector scanner (Canon, Ōta, Japan) with (e.g., imaging cervical abscess) or without (e.g., imaging mastoiditis) contrast agent. The scans were analyzed for presence of complications concerning the upper respiratory tract by a radiologist (C.M.) with 5 years of experience in MRI and 1 year of experience in pediatric radiology, who was aware of the clinical information and of the initial reports. In cases of disagreement, these cases were evaluated by consensus with the radiology specialists who had provided the clinical report and had at least 7 years of imaging experience, some of whom were subspecialists in pediatric radiology.

Data were compared to MRI and CT scans performed in the same months of the preceding five winters (October to April of 2017/2018, 2018/2019, 2019/2020, 2020/2021, and 2021/2022), distinguishing between pre-pandemic (2017/2018, 2018/2019, 2019/2020) and pandemic years (2020/2021, 2021/2022). Further information was collected from the medical records including: results of pathogen testing, inflammatory serum markers (C-reactive protein (CRP) and leukocytosis), and surgery reports, if surgery was performed.

### Statistics

Statistical tests were performed using SPSS Version 25 (IBM, Armonk, NY), Excel 2016 (Microsoft, Redmond, WA), and R Version 4.3.0 (R Core Team, Auckland, New Zealand). Poisson distribution was used to test differences between the assessed numbers and rates of a certain winter season and the expected number based on the other included winter seasons. Continuous variables are reported as mean ± standard deviation. Statistical significance was set at *P*<0.05.

## Results

Table 1 shows a detailed summary of data obtained for each winter season.

**Table 1:** Summary of data obtained for each winter season. Indicated *P*-values refer to the comparison between the 2022/2023 season and the preceding five years

	10/2017–04/2018	10/2018–04/2019	10/2019–04/2020	10/2020–04/2021	10/2021–04/2022	10/2022–04/2023	*P*
Number of cases							
MRI	12	15	20	12	22	45	<0.001
CT	9	15	10	6	15	44	<0.001
MRI and CT	4	9	5	2	2	23	<0.001
Total	25	39	35	20	39	112	<0.001
Age (y)							
Mean ± std	7 ± 6	7 ± 8	8 ± 6	11 ± 5	7 ± 5	6 ± 5	0.122
Median	6	6	5	12.5	6	5.5	0.144
Sex (f/m) (rate of female in %)	11/14 (f, 44%)	17/22 (f, 44%)	13/22 (f, 37%)	10/10 (f, 50%)	18/21 (f, 46%)	55/57 (f, 49%)	0.013 (0.044)
Number of complications							
Mastoiditis	10	12	7	1	10	45	<0.001
Phlegmon	5	11	10	3	12	31	<0.001
Abscess	11	17	15	8	23	59	<0.001
Meningitis	0	2	0	2	0	8	<0.001
Reactive vasculitis	0	0	0	0	0	3	<0.001
Sinus vein thrombosis	1	1	0	3	0	8	<0.001
Total (rate in %)	19 (76)	30 (77)	27(77)	12 (60)	34 (87)	103 (92)	<0.001 (0.008)
Surgery							
Available data (rate in %)	25 (100)	39 (100)	35 (100)	20 (100)	39 (100)	112 (100)	<0.001 (1.000)
Surgery (rate in %)	16 (64)	28 (72)	26 (74)	12 (60)	31 (80)	90 (80)	<0.001 (0.023)
Pathogen testing							
Available data (rate in %)	17 (68)	24 (62)	30 (86)	15 (75)	35 (90)	102 (91)	<0.001 (0.011)
Detected bacteria incl. streptococci (rate in %)	12 (71)	16 (67)	20 (65)	10 (67)	22 (63)	68 (67)	<0.001 (0.053)
Detected streptococci (rate in %)	8 (67)	11 (69)	14 (70)	4 (40)	14 (64)	52 (76)	<0.001 (0.010)
Serum parameters							
Available data (rate in %)	25 (100)	33 (85)	35 (100)	20 (100)	36 (92)	97 (87)	<0.001 (0.029)
CRP (mean ± std, mg/l)	54 ± 49	76 ± 60	67 ± 72	104 ± 51	80 ± 73	80 ± 66	0.041
Leukocytosis (mean ± std, /nl)	15 ± 7	15 ± 7	14 ± 7	13 ± 6	15 ± 8	15 ± 7	0.101

*CRP* C-reactive protein, *CT* computed tomography, *f *female, *m *male, *MRI* magnetic resonance imaging

During the 2022/2023 winter season, 45 children with upper respiratory tract infection underwent MRI and 44 had a CT scan. Both MRI and CT examinations were performed in 23 patients, resulting in a total of 112 patients (57 males; mean age 6 ± 5 years, median age 5.5 years), who were included in the study.

These 112 cases of cross-sectional imaging in children with upper respiratory tract infections in the 2022/2023 winter season were significantly more than expected based on the data from the winter seasons of the preceding five years, in which the respective mean was 32 cases (*P*<0.001). The following complications were detected: mastoiditis, abscess, phlegmon, meningitis, reactive vasculitis, and sinus vein thrombosis. Abscesses were predominantly located periauricular, intracranial, and periorbital. Phlegmons were diagnosed using MRI and were, by far, most frequently located in the orbit/periorbital area, occasionally in cervical or other facial localizations, such as the nose or cheek. Reactive vasculitis, which was diagnosed in MRI, affected the petrous and cavernous segment of the internal carotid artery characterized by vessel wall thickening and consecutive luminal narrowing. Sinus vein thrombosis was found in the sigmoid and transverse sinuses and partly extended into the internal jugular vein.

The number and rate of complications diagnosed by imaging were also significantly higher than expected based on the data from previous winter seasons, with a complication rate of 92% (mean of prior years, 75%; *P*<0.001). The complication rate was higher at younger ages with 98% in children younger than 4 years, 95% in children under 7 years, and 87% from 7 years old (*P*=0.026).

**Fig. 1:**
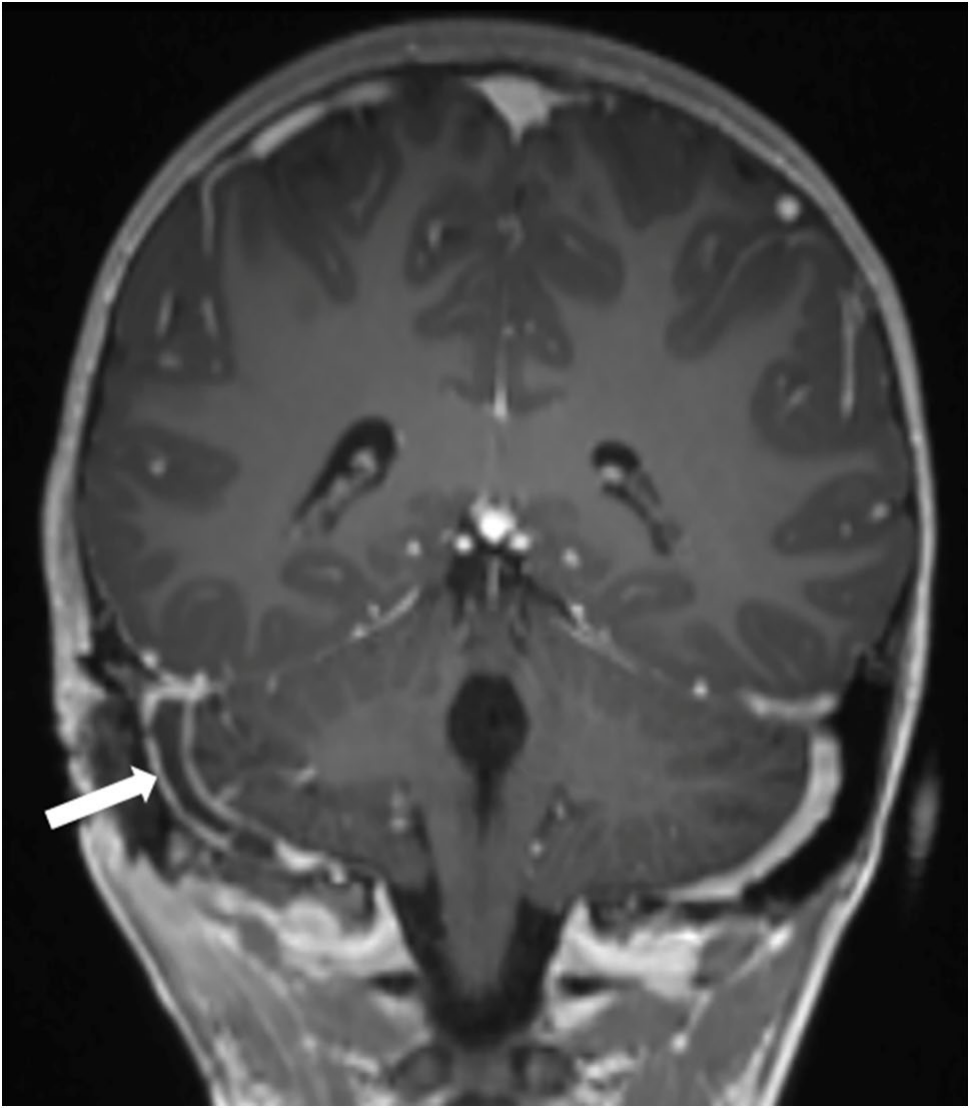
Coronal T1-weighted magnetization prepared-rapid gradient echo sequence magnetic resonance image after intravenous contrast agent administration in a 5-year-old girl with a sinus vein thrombosis in the right sigmoid sinus (*arrow*) caused by mastoiditis

**Fig. 2:**
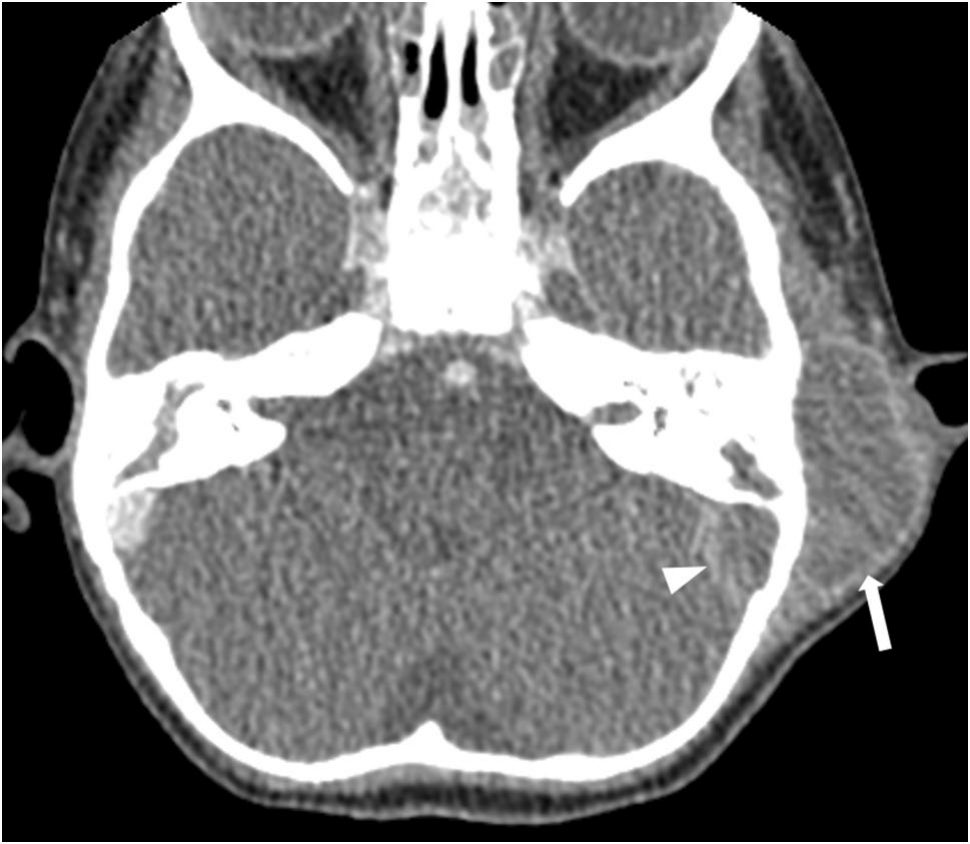
Axial post-contrast computed tomography image (soft tissue window) in a 2-year-old girl with a sinus vein thrombosis in the left sigmoid sinus (*arrowhead*) and a retroauricular abscess (*arrow*) caused by mastoiditis

**Fig. 3:**
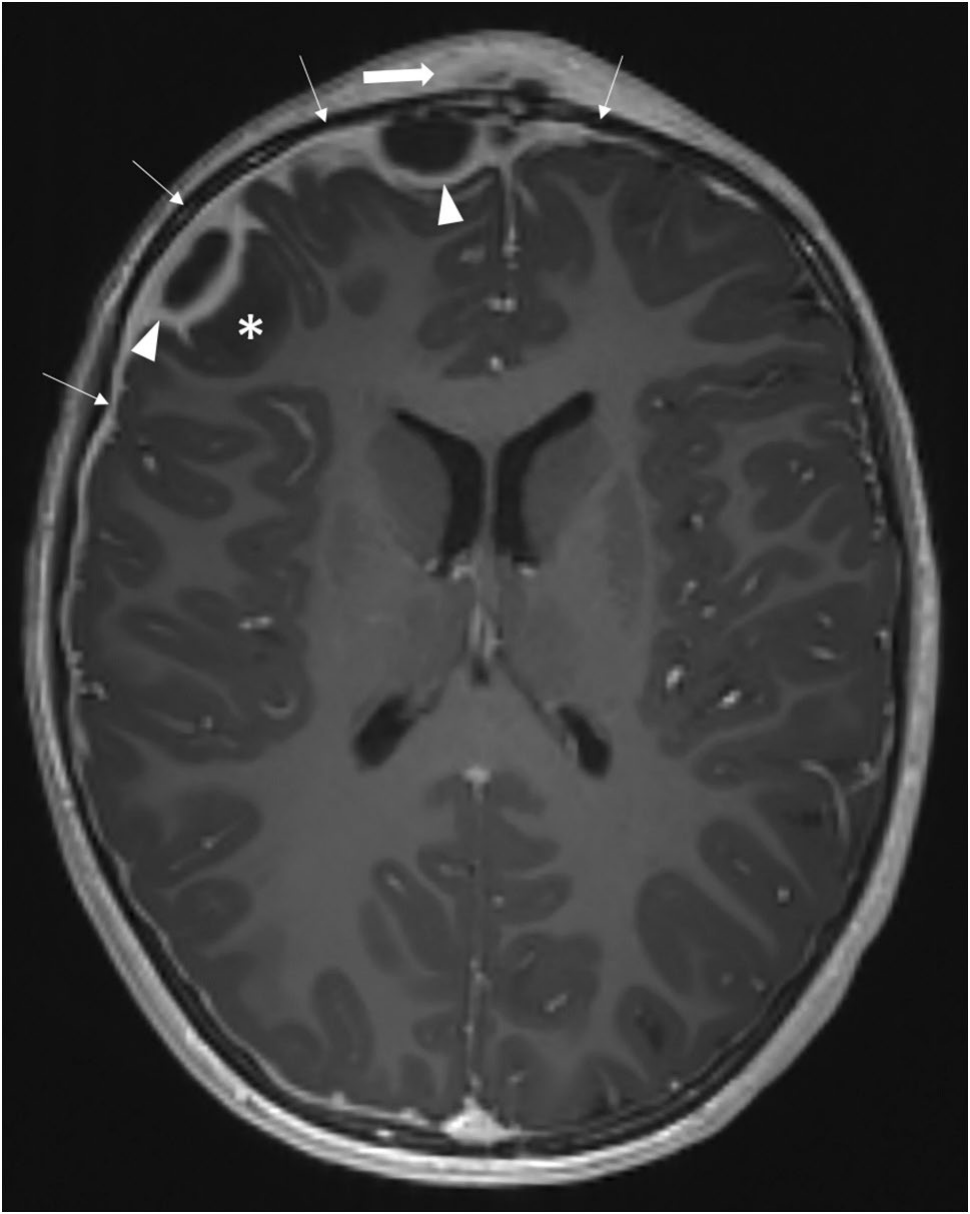
Axial T1-weighted magnetization prepared-rapid gradient echo sequence magnetic resonance image after intravenous contrast agent administration in an 8-year-old boy with osteomyelitis of the frontal bone as a complication of acute sinusitis of the frontal sinus and ethmoidal cells. Subperiosteal abscess on the forehead (Pott’s puffy tumor, *thick arrow*) and frontal epidural and subdural abscesses (*arrowheads*) are visible as well as accompanying cerebritis of the right-sided gyrus frontalis inferior (*asterisk*) and accompanying meningeal reaction (*thin arrows*)

**Fig. 4:**
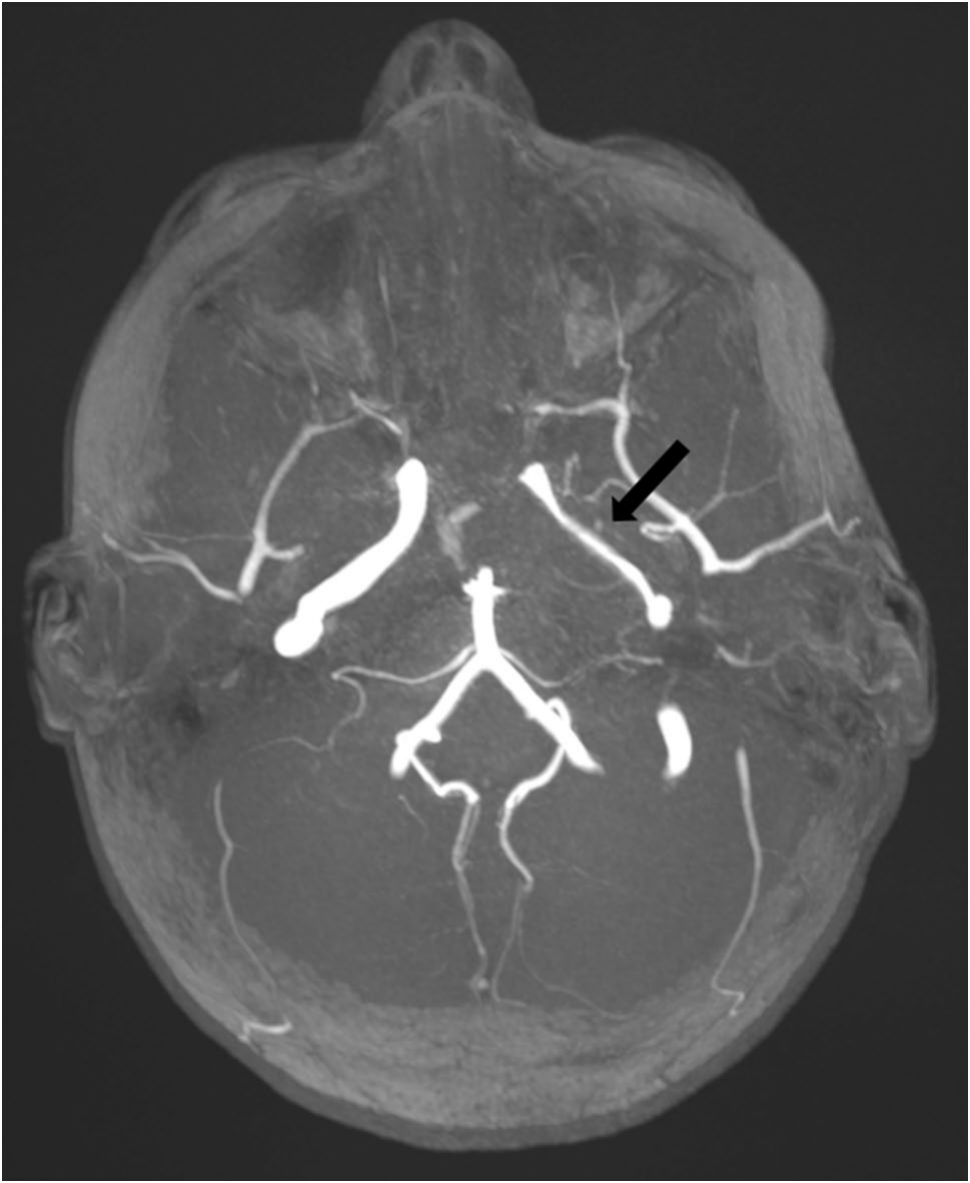
Axial multi-slab of a time-of-flight angiography 3-dimensional sequence magnetic resonance image without contrast agent administration in a 5-year-old girl with extensive abscess at the left skull base with reactive vasculitis of the internal carotid artery in the petrous part M1 (*arrow*) as a complication of otitis media and mastoiditis

**Fig. 5:**
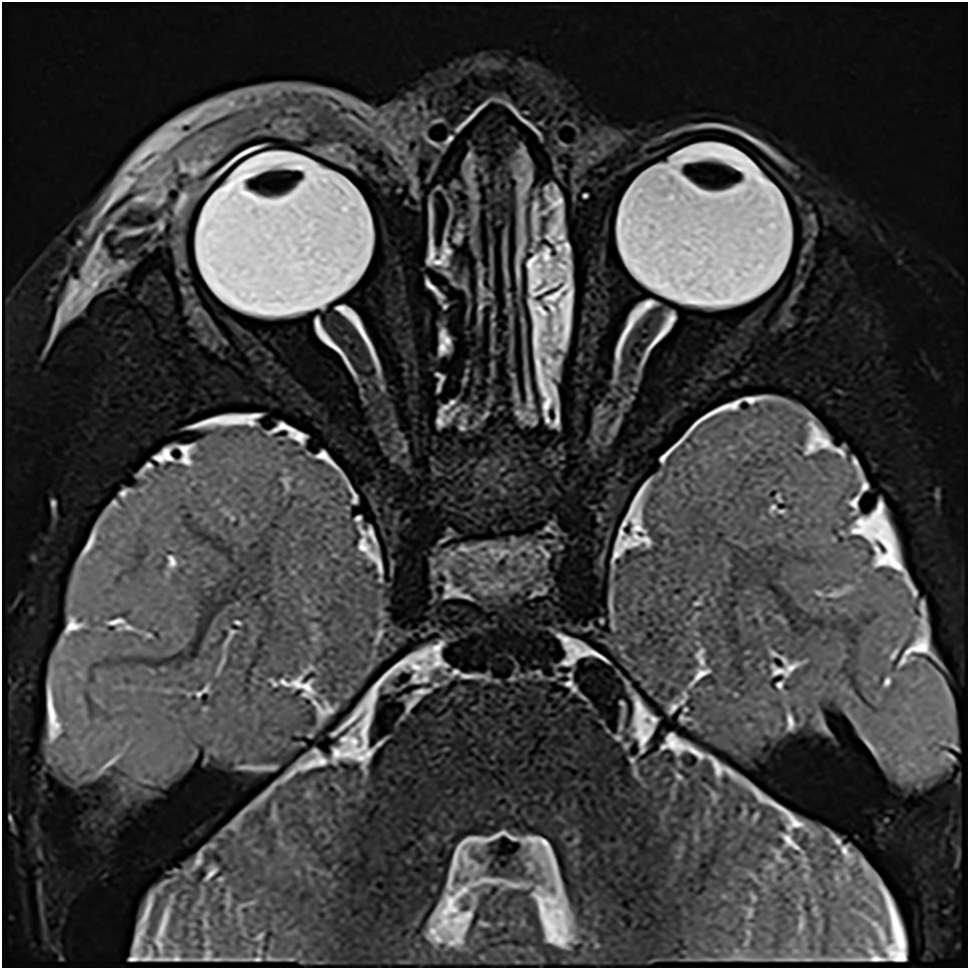
Axial T2-weighted turbo-spin-echo sequence magnetic resonance image with fat-saturation without contrast agent administration in a 2-year-old girl with a right-sided periorbital phlegmon as a complication of acute sinusitis

**Fig. 6:**
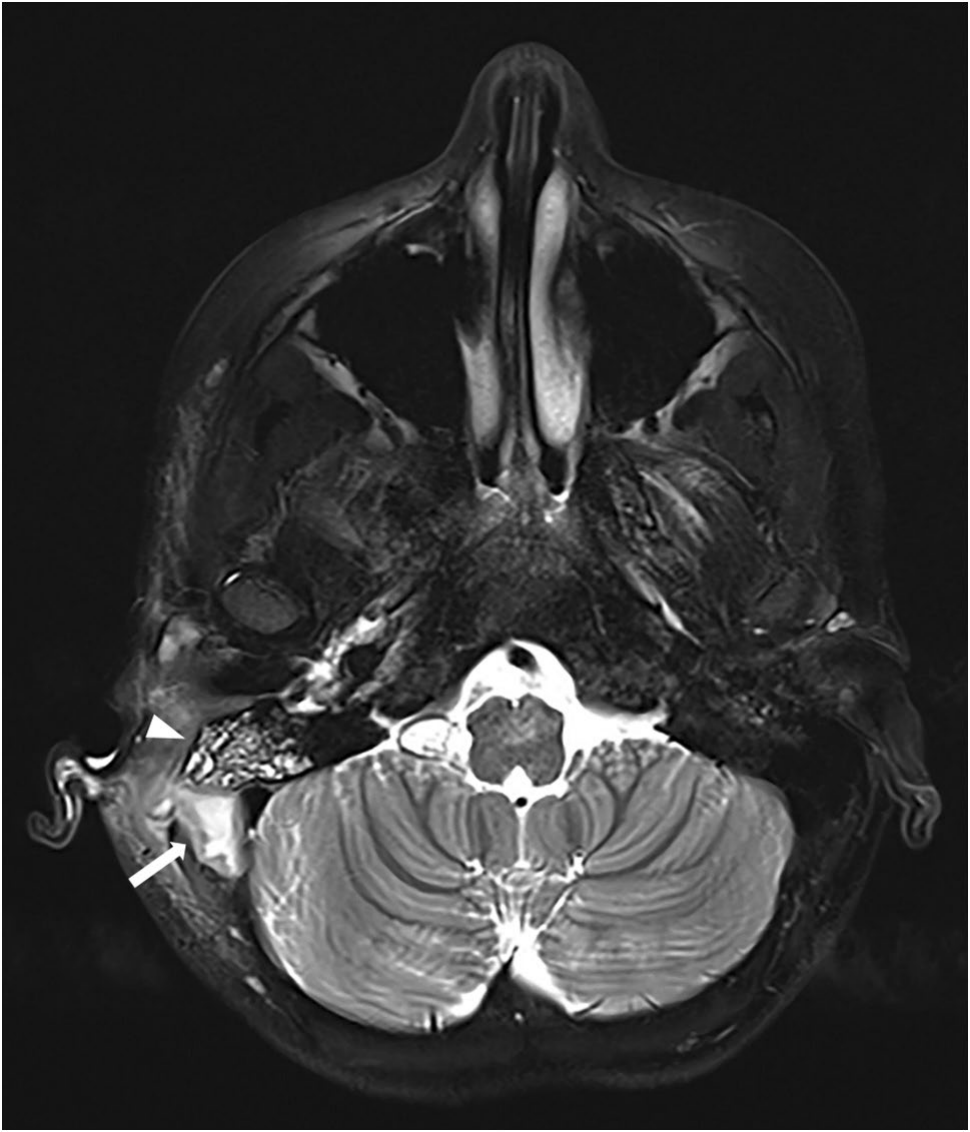
Axial T2-weighted turbo-spin-echo sequence magnetic resonance image with fat-saturation without contrast agent administration in an 8-year-old girl with mastoiditis (*arrowhead*). There is a concomitant abscess (*arrow*) on the right

**Fig. 7:**
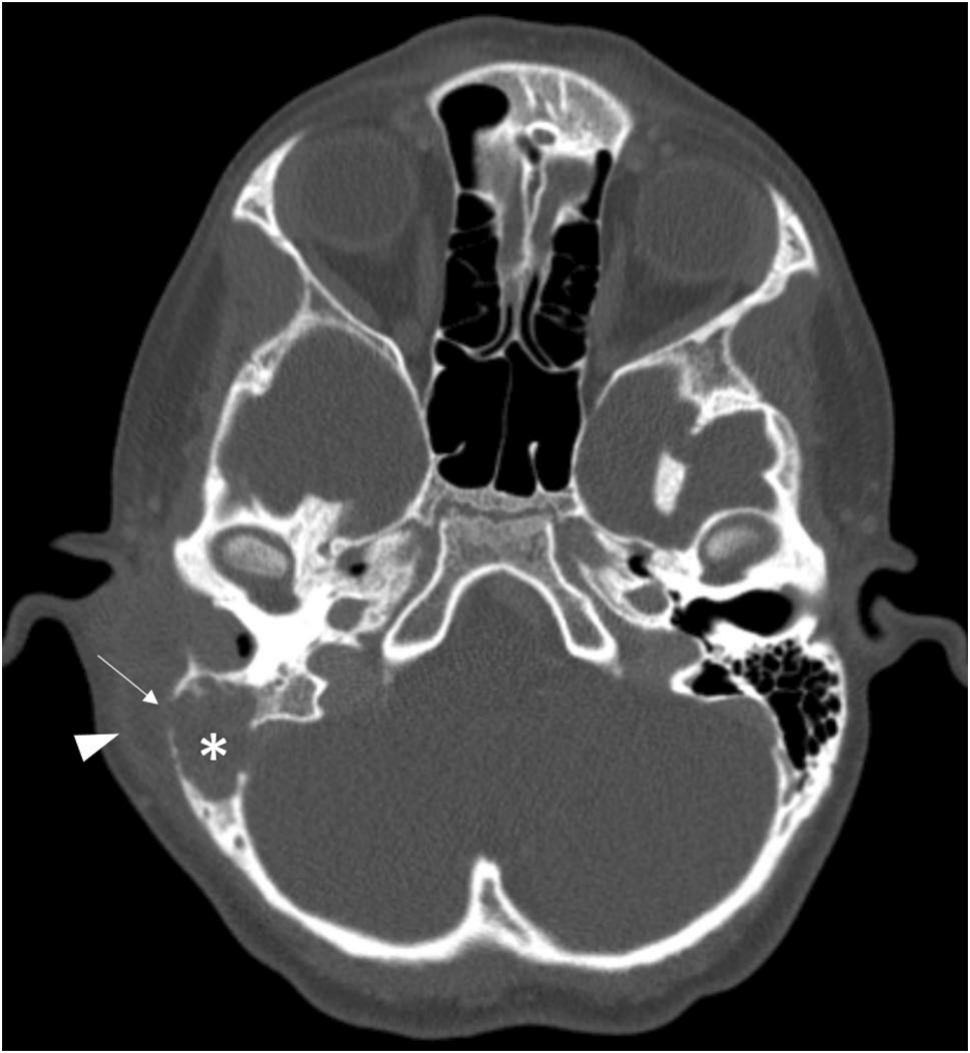
Axial computed tomography image (bone window) without contrast agent administration in a 13-year-old boy with right mastoiditis (*asterisk*) and osseous destruction (*arrow*) and extraosseous periauricular abscess (*arrrowhead*)

**Fig. 8:**
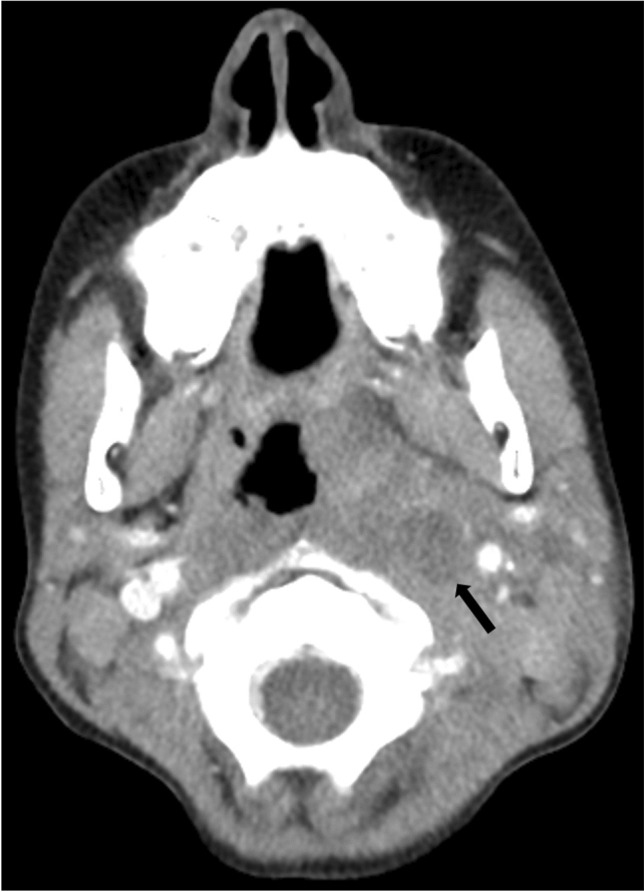
Axial computed tomography image (soft tissue window) without contrast agent administration in a 7-year-old boy with a left retrotonsillar abscess due to tonsillitis (*arrow*)

Each of the complications assessed showed a significant increase during the winter season of 2022/2023. Severe complications especially increased during the first post-pandemic winter season. For example, vascular complications (sinus vein thrombosis and reactive vasculitis) increased from only five cases within the prior five years to 11 cases during the 2022/2023 winter season. Reactive meningitis as a complication of upper respiratory tract infection increased from four cases in the prior five winter seasons to eight cases during the post-pandemic winter season. Table 1 provides an overview of the number of cases of each imaging-based complication recorded per winter season. Figures 1, 2, 3, 4, 5, 6, 7, and 8 show examples of each of the assessed complications from the 2022/2023 winter season.

Regarding the results of pathogen testing, the rate of detected bacterial infections did not significantly differ within the 6-year period studied (*P*=0.053). Streptococci (especially, *Streptococcus pyogenes, intermedius, pneumoniae, constellatus*) made up the majority followed by staphylococci (particularly *Staphylococcus aureus, epidermidis, and auricularis*) and bacillus species. However, the rate of cases with detected bacterial infection with streptococcal species in 2022/2023 winter season was 76%, significantly higher than it was expected based on the prior five winter seasons with a mean rate of 62% (*P*=0.010). Complications arose in all confirmed streptococcal infections and streptococci were detected in most serious complications, such as abscesses and intracranial difficulties. There was also a significant increase in the rate of surgical treatment, including functional endoscopic sinus surgery, mastoidectomy, adenectomy, paracentesis with insertion of a tympanic drainage, abscess clearage (intracranial, cervical), tonsillectomy, and combinations thereof, in pediatric cases with complications of upper respiratory tract infections visualized by MRI and CT (*P*=0.023).

During the first complete pandemic winter season in Europe (2020/2021), the number of MRI and CT scans in children with upper airway infections decreased significantly to 20 cases compared to a mean of 33 cases in the pre-pandemic years, 39 cases in the second pandemic year, and 112 cases in the first post-pandemic winter season (*P*<0.001). Additionally, the complication rate (*P*=0.002), the rate of detected streptococcal infections (*P*<0.001), and the rate of surgery (*P*=0.012) decreased significantly in the 2020/2021 winter season compared to the other winter seasons assessed. In the second pandemic winter season, the numbers and rates recovered to pre-pandemic levels.

The CRP level was lowest in 2017/2018 and highest in 2020/2021, which is a statistical significant difference (*P*<0.001). Leukocytosis did not differ significantly between the years (*P*=0.101).

## Discussion

In this study, we performed an imaging-based analysis of the development of complicated upper respiratory tract infections during and after the COVID-19 pandemic in Germany.

In the first post-pandemic winter season of 2022/2023, a significant increase in imaging for the evaluation of complicated upper respiratory tract infections in children was noted. The number of performed pediatric scans as well as the complication rate, the rate of detected streptococcal infections, and the number of surgeries each increased significantly compared to pre-pandemic and pandemic years. Recent studies suggest that the increased incidence of infectious disease following the pandemic is likely due to reduced individual immunity and lower herd immunity resulting from strict measures to prevent respiratory infections and hospitalizations during the pandemic [22]. This assumption is supported by the result that younger children (≤ 3 years) were affected more frequently and had a higher complication rate, probably because they were born close to the pandemic and were therefore unable to build up a sufficient immune defense. These same measures can be expected to have led to fewer hospital admissions, and thus to a reduced number of pediatric imaging investigations performed, and lower rates of complications, streptococcal infections, and surgery in children with upper respiratory tract infections, which was observed in the first complete pandemic winter season of 2020/2021 in Europe. Similar results were reported by Perniciaro et al., who found an increase of invasive pneumococcal disease in spring and summer of 2021 following a sharp decline in 2020 [23]. Another study performed in China reported a significantly increased number of cases of sinusitis in children after the pandemic and a significantly decreased number of cases of sinusitis during the pandemic, each compared to pre-pandemic years [24]. A Canadian study showed a decrease of pneumococcal infections during the pandemic and a post-pandemic increase [25]. The present study adds to the existing knowledge that not only did the number of imaging studies in children with upper respiratory tract infections increase rapidly after the pandemic, but that these infections also resulted in more complications and that the number of infections caused by streptococcus species increased significantly. Streptococci are pathogens often seen in cohorts of children with abscesses in the head and neck region [26, 27].

In particular, severe complications occurred more frequently during the 2022/2023 winter season. This could also be due to reduced pathogen contact during the pandemic, possibly contributing to a delayed immune reaction due to reduced/missing memory cells for current pathogens [28] or due to pathogen- and type-specific changes as it was suspected by Steens et al. [4].

The increase in available pathogen testing data can be attributed to an increase in complication rates, as it can be assumed that pathogen testing would be performed more frequently during a period with many complications to optimize treatment and prevent severe disease progression. Further, with the increase in surgery rate, the number of intraoperative samples for germ detection increases as testing is regularly performed during such procedures.

This study has limitations. First, since it is retrospective, information on vaccination status against SARS-CoV-2 or whether and when infection with COVID-19 was encountered is missing. The effects of this information could be of significance. The time interval between symptom onset and imaging is inherently inconsistent and depends on the timing of the physician consultation and the decision of the treating physician to perform imaging. We assume that the clinical management of patients with upper respiratory tract infection was identical before, during, and after the pandemic, and thus the time intervals between symptom onset and imaging were comparable. It is conceivable that during the pandemic there was a longer interval between symptom onset and physician consultation, as patients were known to be reluctant to seek hospital care during the pandemic. However, our data show that there were no increased rates of complications during the pandemic because of such delays in diagnosis.

The probability of a pandemic with similar impact to COVID-19 is approximately 2% in any given year and it is suspected that this will increase, underlining the need for further investigation of secondary risks, such as increases in post-pandemic infections in the population [29, 30]. It remains to be observed how the infection situation will evolve in the coming winter seasons. Further investigations to assess differences in post-pandemic infection rates between countries with different preventing measurements during the pandemic might be of interest.

## Conclusion

After a decline during the first pandemic winter season in 2020/2021, there was an immediate rebound in complicated upper respiratory tract infections to pre-pandemic levels in children during the second pandemic winter season. However, in the post-pandemic winter season of 2022/2023, a significant increase in cases and associated complications was observed, compared with pre-pandemic levels. Most likely, this increase was due to immunologic susceptibility resulting from reduced contact with pathogens during the pandemic. Increased rates of severe upper respiratory tract infection following a pandemic should be considered by clinicians so that they can identify complicated cases at an early stage and by managers when planning adjustments of treatment capacity for similar future situations.

## Data Availability

The datasets generated during and/or analyzed during the current study are available from the corresponding author on reasonable request.
